# An efficient protocol for regenerating shoots from paper mulberry (*Broussonetia papyrifera*) leaf explants

**DOI:** 10.1515/biol-2020-0034

**Published:** 2020-06-10

**Authors:** Siming Cui, Ying Ren, Yahan Hao, Junjie Zhang, Zhouchao Chen, Jintuo Zou, Wei Zhou, Xiaoyang Chen

**Affiliations:** State Key Laboratory for Conservation and Utilization of Subtropical Agro-bioresources (South China Agricultural University), Guangdong Key Laboratory for Innovative Development and Utilization of Forest Plant Germplasm, Guangdong Province Research Center of Woody Forage Engineering Technology, College of Forestry and Landscape Architecture, South China Agricultural University, Guangzhou 510642, China

**Keywords:** paper mulberry, leaf explant, plant regeneration, explant age, inoculation orientation

## Abstract

Paper mulberry (*Broussonetia papyrifera*) is a tree species that has many economic, ecological, and social uses. This study developed an efficient protocol for regenerating shoots from leaf explants using Murashige and Skoog (MS) medium supplemented with different concentrations of plant growth regulators (PGRs), which play vital roles in shoot regeneration. The best result, 86.67% induction frequency and 4.35 shoots per explant, was obtained in the MS medium containing 2.0 mg/L *N*6-benzyladenine (BA) and 0.05 mg/L indole-3-butyric acid. The effects of explant age, orientation, and genotype were also investigated. Explants from young leaves had a greater regeneration frequency than those from old leaves, and the results were better when the distal end of the leaf explant contacted the medium versus the proximal end. Approximately 70.96% of the shoots rooted well in the MS medium containing 0.4 mg/L α-naphthalene acetic acid (NAA). Although some genotypes achieved poorer results, the regeneration protocol is still applicable for mass multiplication and genetic transformation.

## 1 Introduction

Paper mulberry (*Broussonetia papyrifera*) is a multipurpose tree or shrub belonging to the Moraceae [1] that mainly grows in eastern Asia and the Pacific islands [2,3,4]. Each organ of the tree species is valuable: bark fiber is an excellent raw material for paper-making and a potential bio-ethanol feedstock [5,6,7] and young shoots have a high feed and nutritional value [8]. There are many medicinal uses for paper mulberry as a source of flavonoids, terpenoids, polyphenols, alkaloids, and other compounds [9,10,11,12]. In summary, paper mulberry has ecological, economic, forage, and medicinal values and has good development prospects. Consequently, paper mulberry is attracting increasing attention worldwide. Paper mulberry has strong stress resistance and environmental adaptability [13,14]; it is drought resistant and has the strong anti-pollution ability [15,16], allowing it to play an important role in ecological restoration [17,18,19].

The standard method of paper mulberry proliferation is seed breeding, but the survival rate is low and uniform seeding is not obtained by seed proliferation [20]. Paper mulberry can also proliferate through root cultivation and cutting propagation [21,22], but the efficiency is relatively low and cannot meet large-scale production needs. Therefore, to meet the demand for large-scale production and achieve efficient, convenient propagation, it is important to establish an efficient regeneration protocol for paper mulberry using a genetic transformation system based on the fundamental molecular and biochemical characteristics of this plant [23]. Several *B. papyrifera* regeneration protocols have been reported: proliferation from stem explants cultured in various plant growth regulator (PGR) combinations [24,25]; shoot regeneration from hypocotyl explants cultured on Murashige and Skoog (MS) medium containing 0.5–1.5 mg/L *N*6-benzyladenine (BA) and 0.1–0.5 mg/L naphthalene acetic acid (NAA) [26,27]; and shoot proliferation from leaf explants cultured on the MS medium supplemented with 1.5–2.0 mg/L BA and 0.05–1.0 mg/L NAA [28,29,30,31]. However, the proliferation efficiency of these systems is low and not satisfactory for establishing an efficient regeneration protocol. The selection of an effective explant is a prerequisite for any plant regeneration protocol [32]. Leaves are abundant and allow for stable gene transfer, but there is no efficient regeneration protocol using leaves as explants. This study explored the effects of PGRs, age, orientation, and genotype on the regeneration efficiency to establish an efficient shoot regeneration protocol from paper mulberry leaf explants for application in mass multiplication and genetic transformation.

## 2 Materials and methods

### 2.1 Plant materials, explant sources, and growth conditions

Seeds were collected from the Guangzhou cultivar in a field at South China Agricultural University (genotypes not included). Seeds were soaked in concentrated sulfuric acid for 9 min and washed with tap water for 5 min to soften the seed coat. After sterilization with 75% (v/v) ethanol for 50 s and 25% (w/v) NaClO for 15 min, the seeds were rinsed four times with sterile water and inoculated onto the MS medium [26]. When the seeds grew to seedlings after 4 weeks, seedling leaves were used as explants for experiments. All cultures were kept under cool white light (approximately 50 µmol/m^2^/s) with a 12 h photoperiod at 24 ± 1°C.

### 2.2 Induction and growth of shoot-buds

To compare the PGRs influencing the culture response, BA (0.0, 1.0, 1.5, 2.0, and 2.5 mg/L), indole-3-butyric acid (IBA; 0.0, 0.01, 0.05, and 0.10 mg/L), and kinetin (KT; 0.0, 0.5, and 1.0 mg/L) were used in combination in the MS basal medium. To investigate the effect of explant age on shoot regeneration, seedlings were divided into three sections from top to bottom: Section 1, top 1–3 leaves; Section 2, 4–6 leaves in the middle; and Section 3, bottom 7–9 leaves. To investigate the effect of explant orientation on shoot regeneration, we performed an experiment to examine the effects of contact of the proximal and distal ends with the medium. Four tree cultivars were used to examine the influence of genotype on shoot regeneration: NC-1, NC-2, TS-1, and ZQ-1. The percentage induction of shoot-buds and the number of shoot-buds per explant were recorded after 5 weeks of culture.

### 2.3 Rooting and acclimatization

The shoots were cut from the tissue and transferred onto the MS basal medium supplemented with NAA (0.0, 0.1, 0.2, 0.4, and 0.8 mg/L) to facilitate root initiation and the growth of intact plantlets. Plantlets with well-developed roots were removed from the culture bottles and washed under running tap water to remove any remaining medium. Then, the plantlets were placed in plastic pots filled with planting substrate in a greenhouse at high humidity. After acclimatization for 3 weeks, the plantlets were ready for transplantation in the field.

### 2.4 Data collection and statistical analysis

The experiments were conducted in a randomized complete block design; each treatment consisted of ten explants and was repeated three times. Analysis of variance (ANOVA) was carried out using SPSS 22.0. Duncan’s multiple range test was used to detect differences among means, with a significance level of 0.05.

## 3 Results and discussion

### 3.1 Effect of PGRs on shoot-bud induction

**Figure 1:**
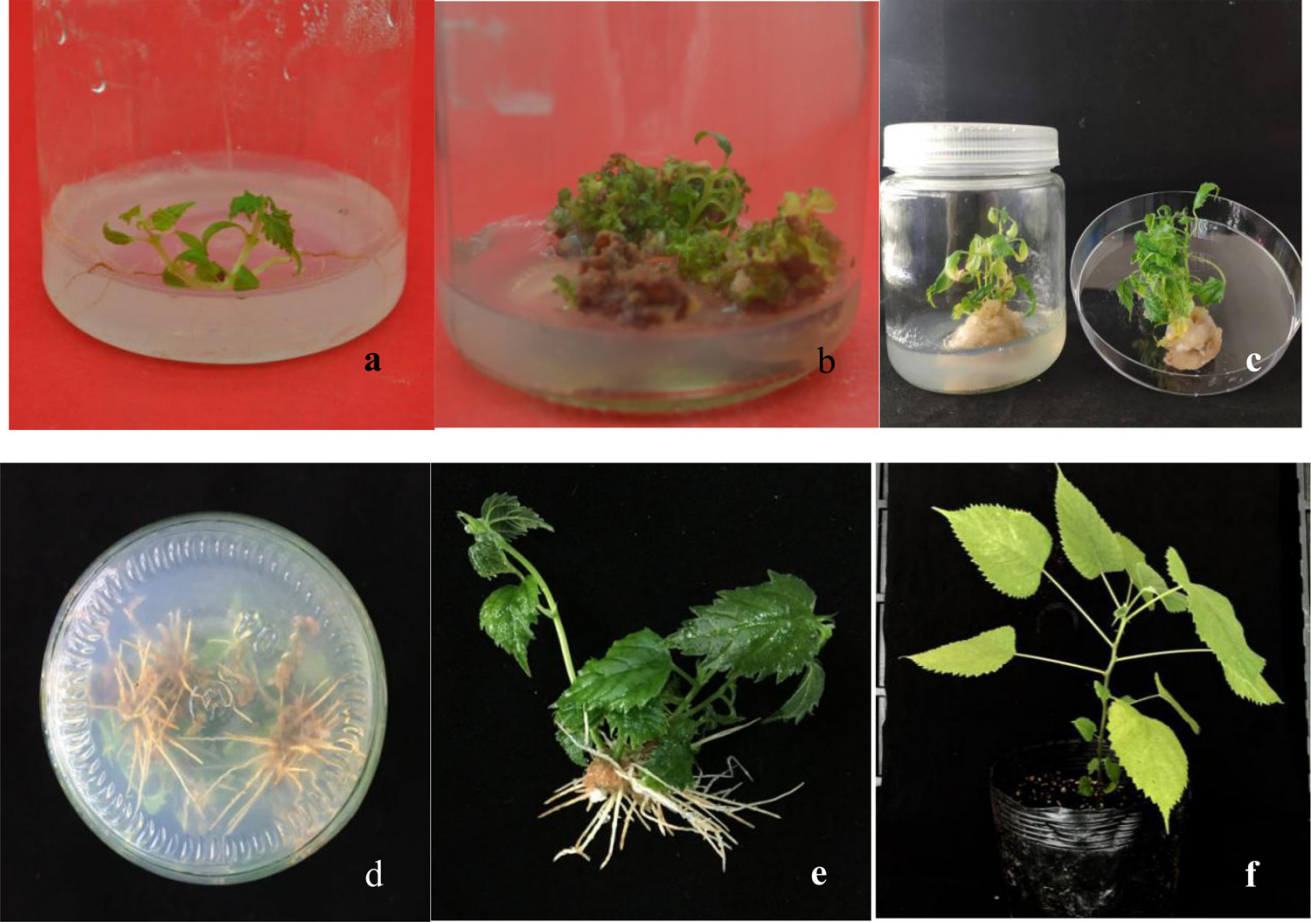
Shoot regeneration from paper mulberry leaf explants and acclimatization of the regenerated plantlets. (a) 2 week-old seedling; (b) numerous shoot buds developed from leaf explants in the MS medium with 2.0 mg/L BA and 0.05 mg/L IBA in 3 weeks; (c) 2–3 cm-high shoots recovered from leaf explants in the MS medium with 2.0 mg/L BA and 0.05 mg/L IBA for 4 weeks; (d) root systems of regenerated shoot buds in the MS medium supplemented with 0.4 mg/L NAA; (e) an intact regenerated plantlet; and (f) an acclimatized plant transferred to a plastic pot filled with planting substrate.

The seedlings (Figure 1a) were removed from the culture bottles aseptically, and only the leaves from Section 1 were used as explants for the experiment. The leaves were excised, cut into 5 × 5 mm^2^ pieces, and inoculated on the MS medium supplemented with various concentrations and combinations of BA, IBA, and KT for shoot induction. In the *in vitro* regeneration process, leaves began to expand gradually, the thickness gradually increased, the color changed from green to light, and the leaves were wrinkled and curled in 1 week. Shoot-buds appeared at 3 weeks (Figure 1b). Only parts of the induced shoot-buds elongated to form shoots.

The experimental results are shown in Tables 1–3. It is clear that PGRs played a vital role in shoot regeneration, and the proliferation at different concentrations was much better than when the PGRs were used alone (Tables 1 and 2). Although BA was more effective at promoting paper mulberry shoot regeneration than IBA, the best results were obtained when they were used together (Tables 1 and 2). KT had an inhibitory effect; the best results were obtained without KT (Table 3). The greatest efficiency was achieved when the MS basal medium was supplemented with 2.0 mg/L BA and 0.05 mg/L IBA: the shoot induction efficiency was 80.33% and there were 4.33 regenerated shoots (Tables 1–3).

**Table 1:** Effect of different BA concentrations on shoot-bud induction

BA (mg/L)	Explants that regenerated shoots (%)	Shoots per explant
0.0	0 e	0 e
1.0	23.33 ± 13.02 cd	2.33 ± 0.26 cd
1.5	50.00 ± 10.86 ab	3.70 ± 0.34 ab
2.0	80.33 ± 11.68 a	4.33 ± 0.56 a
2.5	36.67 ± 12.51 bc	3.40 ± 0.42 bc

Each value represents the mean ± standard error of three replicates, each with 10 explants. The medium contained 0.05 mg/L IBA and 0.0 mg/L KT. Means followed by the same letter in the same column are not significantly different from each other at *P* ≤ 0.05, according to Duncan’s multiple range test.

**Table 2:** Effect of different IBA concentrations on shoot-bud induction

IBA (mg/L)	Explants that regenerated shoots (%)	Shoots per explant
0.00	13.33 ± 9.08 b	2.73 ± 0.36 b
0.01	56.67 ± 13.33 ab	3.57 ± 0.41 ab
0.05	80.33 ± 11.68 a	4.33 ± 0.46 a
0.10	10.00 ± 8.51 c	2.87 ± 0.26 c

Each value represents the mean ± standard error of three replicates, each with 10 explants. The medium contained 2.0 mg/L BA and 0.0 mg/L KT. Means followed by the same letter in the same column are not significantly different from each other at *P* ≤ 0.05, according to Duncan’s multiple range test.

**Table 3:** Effect of different KT concentrations on shoot-bud induction

KT (mg/L)	Explants that regenerated shoots (%)	Shoots per explant
0.0	80.33 ± 11.68 a	4.33 ± 0.56 a
0.5	46.33 ± 12.98 b	2.93 ± 0.34 b
1.0	26.67 ± 11.01 b	1.87 ± 0.25 b

Each value represents the mean ± standard error of three replicates, each with 10 explants. The medium contained 2.0 mg/L BA and 0.05 mg/L IBA. Means followed by the same letter in the same column are not significantly different from each other at *P* ≤ 0.05, according to Duncan’s multiple range test.

Several regeneration protocols using leaves for explants have been reported for *B. papyrifera*. For example, Huang et al. [31] found that culturing paper mulberry leaf explants on the MS medium supplemented with 2.0 mg/L BA and 0.1 mg/L NAA had the best induction efficiency (81.7%), the regeneration efficiency was similar to this experiment, but the results did not show the number of shoots per explant, which was not rigorous. Liu et al. [30] found that regenerating shoots with leaf explants on the MS medium containing 1.5 mg/L BA and 1.0 mg/L NAA created the maximum number of shoots (3.88 shoots per explant), which was less than this experiment, and the experimental results did not show the regeneration efficiency, which was imperfect. However, Yu et al. [28] achieved a shoot induction efficiency of 36.4% propagating paper mulberry using leaf explants only, which was low. The effects of BA in organogenesis are supported by similar reports in other plants [33,34]. These results suggest that cell activities during shoot-bud regeneration are controlled by various internal factors including cytokinins, which may cause internal chemical and structural differences, leading to different responses when present in the medium. It is worth mentioning that NAA was used for auxin in the above studies, and IBA was used in this study. Obviously, the results of this study are better, and similar results have been reported in hemp [35]. However, the specific roles of BA and IBA remain unclear [26].

### 3.2 Effect of explant age on shoot-bud regeneration

Explant age affected the frequency of shoot-bud regeneration and the mean number of shoots per explant (Table 4). Explants from Section 1 had the greatest regeneration frequency (86.67%) and produced 4.35 shoot buds per explant. Therefore, the explant age plays an important role in shoot regeneration. Similar results were obtained in other plants. For example, Vaidya et al. [36] found that the age of peppermint shoot tip explants affected regeneration frequency; Singh [37] observed that the regeneration efficiency of *Jatropha curcas* was affected by the age of leaf explants; explants from younger seedlings (≤15 days) were still juvenile and formed callus easily, whereas beyond 30 days, the regeneration response decreased with an increase in the age of seedlings. Younger explants have greater morphogenic potential than older explants, as they might have more metabolically active cells due to hormonal, nutritional, and other physiological conditions that are responsible for increased organogenesis [33]. However, Zhang et al. [23] found that for Moringa, the explants were either too young or too old with unsatisfactory results. This may be due to the different meristematic capabilities of different plants. Each plant only has enough metabolically active cells if it grows to a specific age.

**Table 4:** Effect of explant age on shoot regeneration and elongation

Leaf age	Explants that regenerated shoots (%)	Shoots per explant
Section 1	86.67 ± 12.33 a	4.35 ± 0.22 a
Section 2	76.67 ± 13.02 a	3.23 ± 0.56 ab
Section 3	60.00 ± 11.82 ab	2.53 ± 0.31 ab

Each value represents the mean ± standard error of three replicates, each with 10 explants. The medium contained 2.0 mg/L BA, 0.05 mg/L IBA, and 0.0 mg/L KT. Means followed by the same letter in the same column do not differ significantly from each other at the *P* ≤ 0.05 level, according to Duncan’s multiple range test.

### 3.3 Effect of explant orientation on shoot-bud propagation

The orientation of the explants significantly influenced the shoot-bud induction response. When the distal end of the leaf explants contacted the medium, the shoot-bud regeneration rate reached 73.33%. However, this rate decreased to 13.67% when the proximal end of the explant contacted the medium. The number of induced shoot buds per explant increased from 1.02 to 3.58 after placing the distal end of the leaf explants contacted the medium (Table 5). In the cultures, shoot buds were mostly induced from the vein and edge of the cut surface of the distal end, as has been reported for sugarcane [38]. Similarly, Kumar et al. [39] found that *Salvadora oleoides* had a higher regeneration efficiency when the distal end of leaf explants contacted the medium. When studying the effect of explant orientation on regeneration efficiency, not only leaf explants, Liu et al. [40] found that for petiole explants of *Jatropha curcas* L., the method of inoculating the explants horizontally on the medium was more beneficial to the regeneration than inoculating the explants vertically. These results may be related to the transport of polar auxin, because the intensity of auxin polar transport is inversely related to the sensitivity of rooting [41]. Moreover, the logical explanation for the effects of orientation of the explants may be the oxygen tension in the meristematic cells at the explant–medium interface [38]. However, the specific reasons are not clear, and further research is needed.

**Table 5:** Effect of explant orientation on shoot propagation and elongation

Orientation	Explants that regenerated shoots (%)	Shoots per explant
Distal end	73.33 ± 14.98	3.58 ± 0.42
Proximal end	13.67 ± 9.91	1.02 ± 0.27

Each value represents the mean ± standard error of three replicates, each with 10 explants. The medium contained 2.0 mg/L BA, 0.05 mg/L IBA, and 0.0 mg/L KT. Means followed by the same letter in the same column do not differ significantly from each other at the *P* ≤ 0.05 level, according to Duncan’s multiple range test.

### 3.4 Effect of explant genotype on shoot-bud regeneration

To examine the adaptability of the regeneration protocol to different paper mulberry trees, four cultivars with different genetic backgrounds were tested and their regeneration efficiencies and numbers of shoot-buds per explant were compared. As shown in Table 6, the regeneration efficiency and number of shoot-buds per explant varied greatly among the genotypes grown under the same conditions. Explants from ZQ-1 and NC-2 had higher regeneration frequencies of 83.33% and 78.58%, respectively, while explants from NC-1 and TS-1 fared much worse, with regeneration frequencies of 56.67% and 46.67%, respectively. The reason for this difference may be that the endogenous hormone levels varied amongst calli derived from different genotypes and explant types [42]. The differential behavior can be related to different mechanisms for control of the endogenous PGRs metabolism and/or contents. The regeneration capacity of calli is strongly correlated with the concentration of endogenous hormone; recently, Kudo et al. [43] suggested that the endogenous hormone levels affect the regeneration ability of calli. Similar results have been reported in other plants, including *Mucuna bracteata* DC. ex Kurz [44], *Punica granatum* L. [45], conifers [46], *J. curcas* [47], and jojoba [48].

**Table 6:** Effect of explant genotype on shoot regeneration and elongation

Genotype	Explants that regenerated shoots (%)	Shoots per explant
NC-1	56.67 ± 13.33 b	3.57 ± 0.41 a
NC-2	78.58 ± 10.69 a	3.88 ± 0.27 a
TS-1	46.67 ± 9.88 b	2.33 ± 0.32 b
ZQ-1	83.33 ± 12.33 a	4.21 ± 0.38 a

Each value represents the mean ± standard error of three replicates, each with 10 explants. The medium contained 2.0 mg/L BA, 0.05 mg/L IBA, and 0.0 mg/L KT. Means followed by the same letter in the same column do not differ significantly from each other at the *P* ≤ 0.05 level, according to Duncan’s multiple range test.

### 3.5 Rooting and acclimatization

Shoots 2–3 cm in height (Figure 1c) were placed on rooting medium after 4 weeks. The percentage of rooting and the number and length of roots of each seedling increased with the concentration of NAA added to the MS basal medium (Table 7). The greatest rooting percentage (Figure 1d and e) achieved was 70.96% when shoots were placed on the MS medium containing 0.4 mg/L NAA. However, roots induced on the MS medium with higher concentrations of NAA (0.8 mg/L) developed more, but shorter, weaker roots. A similar observation has been observed in *Simmondsia chinensis* [49].

**Table 7:** Effect of NAA on root induction and growth

NAA (mg/L)	Rooting (%)	Root no.	Root length (cm)
0.0	10.33 ± 8.25 c	3.56 ± 0.42 c	0.71 ± 0.15 b
0.1	50.28 ± 12.33 b	2.82 ± 0.25 c	1.13 ± 0.18 ab
0.2	70.23 ± 11.28 a	5.33 ± 1.58 bc	1.28 ± 0.23 a
0.4	70.96 ± 10.56 a	10.68 ± 1.33 b	1.75 ± 0.31 a
0.8	50.34 ± 12.48 b	16.53 ± 1.54 a	0.73 ± 0.18 b

Each value represents the mean ± standard error of three replicates, each with 10 explants. Means followed by the same letter in the same column do not differ significantly from each other at the *P* ≤ 0.05 level, according to Duncan’s multiple range test.

The rooted shoots survived well after transfer to plastic pots filled with planting substrate (Figure 1f). The acclimatization of the rooted shoots was accomplished and more than 90% of the plants were successfully transferred to plastic pots in greenhouse conditions. The results indicated that the regenerated shoots had good environmental adaptability. Similar results have also been obtained in other plants. Cheng *et al*. [35] found that 75% of the hemp-rooted shoots survived after acclimation. Similarly, Liu et al. [40] observed that more than 80% of *J. curcas* were successfully transferred to plastic pots. These results suggest that most of the regenerated seedlings can survive well after transfer to plastic pots, but the survival rate of different plants is different, probably because their environmental adaptability is different.

## 4 Conclusions

An efficient shoot regeneration protocol from *B. papyrifera* leaf explants was established. The greatest shoot induction efficiency was 86.67%, and the maximum number of shoots regenerated was 4.35. The plants produced with this protocol were healthy and had good environmental adaptability. This method will be useful for subsequent application in mass multiplication and genetic transformation.
